# Reconstruction of cysteine biosynthesis using engineered cysteine-free enzymes

**DOI:** 10.1038/s41598-018-19920-y

**Published:** 2018-01-29

**Authors:** Kosuke Fujishima, Kendrick M. Wang, Jesse A. Palmer, Nozomi Abe, Kenji Nakahigashi, Drew Endy, Lynn J. Rothschild

**Affiliations:** 1 Earth-Life Science Institute, Tokyo Institute of Technology, Tokyo, 1528550 Japan; 2 Universities Space Research Association, NASA Ames Research Center, Moffett Field, California 94035 USA; 3 Institute for Advanced Biosciences, Keio University, Tsuruoka, 9970035 Japan; 4 Stanford University Department of Bioengineering, Stanford, California 94305 USA; 5 Spiber Inc. 234-1 Mizukami, Kakuganji, Tsuruoka 9970052 Japan; 6 NASA Ames Research Center, Moffett Field, California 94035 USA

## Abstract

Amino acid biosynthesis pathways observed in nature typically require enzymes that are made with the amino acids they produce. For example, *Escherichia coli* produces cysteine from serine via two enzymes that contain cysteine: serine acetyltransferase (CysE) and O-acetylserine sulfhydrylase (CysK/CysM). To solve this chicken-and-egg problem, we substituted alternate amino acids in CysE, CysK and CysM for cysteine and methionine, which are the only two sulfur-containing proteinogenic amino acids. Using a cysteine-dependent auxotrophic *E. coli* strain, CysE function was rescued by cysteine-free and methionine-deficient enzymes, and CysM function was rescued by cysteine-free enzymes. CysK function, however, was not rescued in either case. Enzymatic assays showed that the enzymes responsible for rescuing the function in CysE and CysM also retained their activities *in vitro*. Additionally, substitution of the two highly conserved methionines in CysM decreased but did not eliminate overall activity. Engineering amino acid biosynthetic enzymes to lack the so-produced amino acids can provide insights into, and perhaps eventually fully recapitulate via a synthetic approach, the biogenesis of biotic amino acids.

## Introduction

How life emerged from an inorganic prebiotic world to become the complex biological systems of today remains a pressing yet unsolved question. One problem within this grand puzzle is how the building blocks for polypeptides, which are the primary catalysts and structural elements for life, originated and perpetuated. Ten of the twenty standard proteinogenic amino acids—glycine, alanine, aspartic acid, glutamic acid, valine, serine, isoleucine, leucine, proline, and threonine—are found in multiple abiotic settings and thus are likely to have been present on prebiotic Earth^1,2^. Some of these amino acids were transported to Earth via meteorites and comets, while others were generated via terrestrial geochemical events such as lightning, volcanism, and hydrothermal syntheses^3^.

While it has been hypothesized that short RNA fragments formed ribozymes to catalyze various chemical reactions^4^, there is no conclusive evidence for ribozymes in proto-metabolism that would have synthesized the entire library of the modern amino acids. It is unclear even if such syntheses pre- or post-dated the origin of RNA. The set of 20 amino acids is realized via chemical reactions enacted by protein enzymes, and in most cases these enzymes contain the amino acids they synthesize. However, it seems likely that in an earlier time polypeptides composed of a limited abiotically-derived set of amino acids might have served as biosynthetic enzymes^5^. Such polypeptides could have played a role in developing and diversifying amino acid biosynthesis pathways.

Drawing upon past work engineering enzymes with reduced sets of amino acids^6,7^, we hypothesized that we could create biosynthetic enzymes that do not contain the amino acid they synthesize. Our aim was to construct such enzymes and demonstrate that it is possible to solve the chicken-and-egg problems found in modern amino acid biosynthesis, at least for the protein components directly involved with the chemical reaction. Here we focused on the biosynthesis of cysteine, an amino acid that plays an important role in metabolism, as well as modern protein structure and function. As a single molecule, cysteine is one of the key substrates providing sulfur for coenzyme A and a methionine biosynthesis pathway^8^. Cysteine is also used to coordinate iron-sulfur cluster prosthetic groups that are found in key biochemical functions such as electron transfer, catalysis and oxygen sensing^9^. Additionally, cysteine is unique among the twenty proteinogenic amino acids in that two residues can form a disulfide bridge, a structure that results from the thiol group of cysteine. Disulfide bridges in the polypeptide chain are able to cross-link two cysteines to form secondary and tertiary structures^10^. Despite the importance all of these functions suggest, cysteine has been one of the poorly identified compounds via prebiotic synthesis^11^.

**Figure 1:**
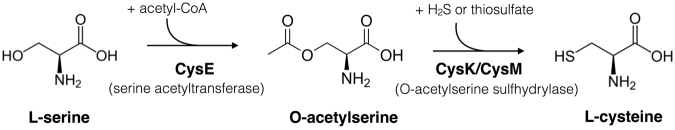
Two step cysteine biosynthesis pathway. The first step of cysteine synthesis is catalyzed by serine acetyltransferase, produced by the gene *cysE*. Serine acetyltransferase converts serine into O-acetylserine. From this step, O-acetylserine sulfhydrylase, produced by gene *cysK*, converts the intermediate into cysteine. O-acetylserine sulfhydrylase function can also be provided by the homologous *cysM* gene. Thus, cysteine production in bacteria requires either *cysE* and *cysK* or *cysE* and *cysM*. In addition, genes similar to *cysE*, *cysK*, and *cysM* can be found within certain archaeal genomes^12^.

In plants and various bacterial species, cysteine is synthesized via a two-step pathway from its precursor L-serine. In *E. coli*, for example, CysE converts serine into O-acetylserine and then CysK or CysM incorporates sulfur from either hydrogen sulfide or thiosulfate to form L-cysteine (Fig. 1). Gene families relevant to *cysE*, *cysK*, and *cysM* genes are also found in certain groups of archaea involved in cysteine synthesis from serine via O-acetylserine or O-phosphoserine precursors^12^,^13^. However, alternative pathways have also been identified. In some heterotrophic species of bacteria and eukaryotes, cysteine is produced via the metabolism of methionine through a transsulfuration pathway^14^. Methanogenic archaea have been known to employ yet another pathway, where O-phosphoserine (Sep) is converted to cysteine via a two-step tRNA-dependent reaction^15^. A recent study shows that the two enzymes involved in this reaction are distributed among diverse uncultured archaea and two groups of bacteria^16^. The existence of these multiple cysteine biosynthesis pathways makes it especially challenging to elucidate the path that lead to the first proteinogenic L-cysteine, and each enzymatic pathway fails to demonstrate a naturally observable cysteine-free alternative. We, therefore, took a direct protein engineering approach to eliminate the end product cysteine from the biosynthetic enzymes corresponding to one of these pathways.

## Results

### Synthetic reconstruction of proteins involved in the cysteine synthesis pathway

**Figure 2:**
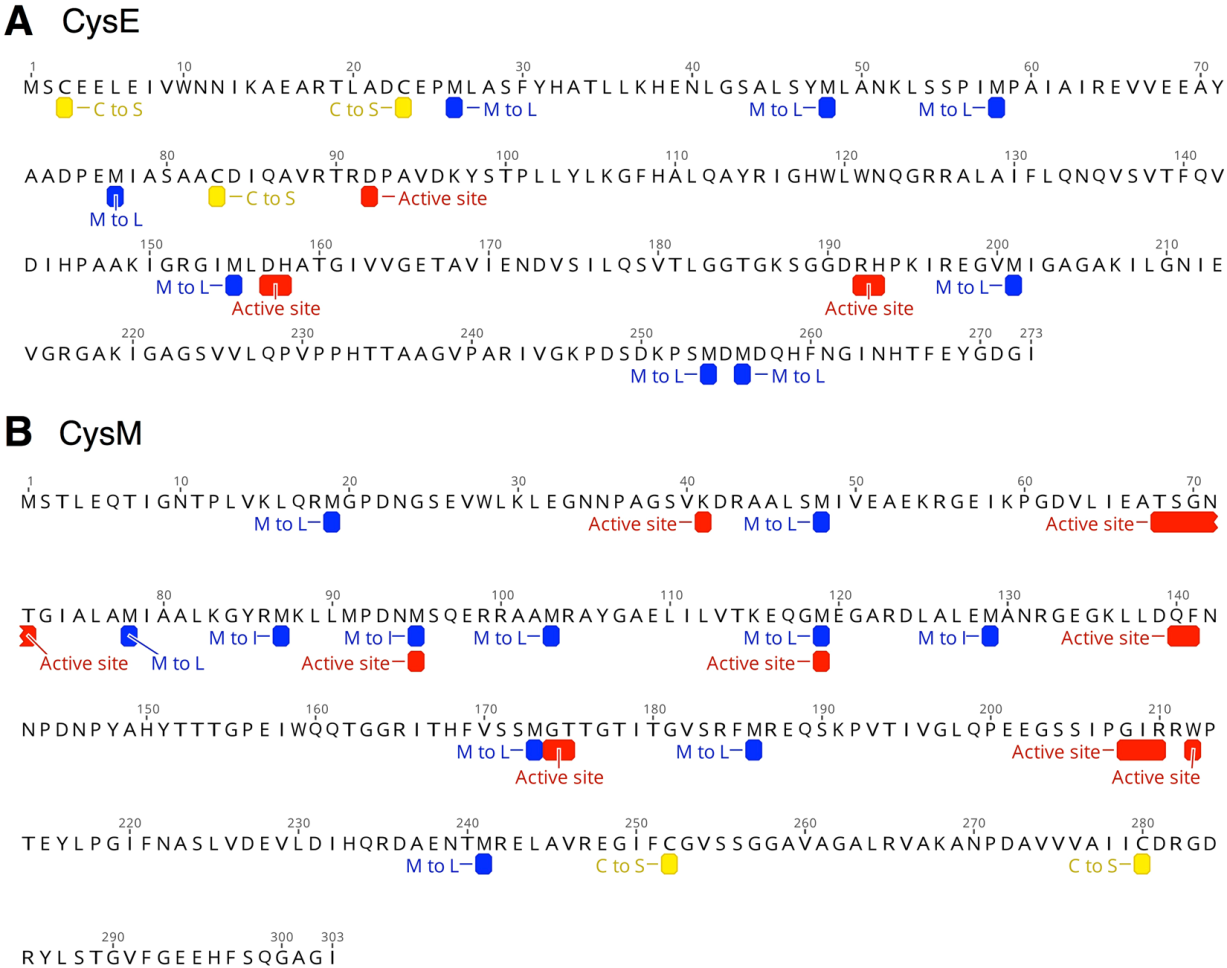
Cysteine and methionine residue substitution sites for c*ysE* and c*ysM* genes. (A) Total of three cysteine residues (3, 23 and 83) and eight methionine residues (26, 48, 58, 77, 155, 201, 254 and 256) were replaced to serine and leucine, respectively. B) Total of two cysteine residues (252, and 280), 11 methionine residues (19, 48, 78, 87, 95, 103, 119, 129, 173, 186 and 241) were replaced to serine and leucine/isoleucine, respectively. ‘C to S’ represents cysteine to serine substitutions (yellow), ‘M to L’ and ‘M to I’ represents methionine to leucine/isoleucine substitutions (blue), and ‘active center’ represents amino acids involved in substrate recognition and catalytic activity of the two enzymes (red).

To solve the chicken-and-egg biosynthetic puzzle for cysteine, we designed a total of four modified synthetic genes (*cysE-C, cysE-CM, cysM-C* and *cysM-CM*) corresponding to either cysteine-free (C) or cysteine-free and methionine-deficient (CM) versions of *E. coli cysE* and *cysM* genes (Fig. 2). Three and two cysteine residues in wild-type *E. coli cysE* and *cysM* genes were changed to serine, respectively. Serine was chosen due to its role as a precursor of cysteine (Fig. 1) as well as its structural resemblance. Additionally, eight and eleven methionine residues were changed to leucine/isoleucine, starting from cysteine-free *cysE-C* and *cysM-C*. The methionine to leucine/isoleucine substitution is thought to be a ‘safe’ substitution that does not disturb protein structure, and results in a similar hydrophobicity to methionine^17^. Synthesized gene sequences were first PCR amplified using primer sequences containing HindIII and XhoI cut sites (Supplementary Fig. S1). Each gene was then cloned into the pUC19 expression vector. Cloned gene sequences were further confirmed based on Sanger sequencing. We also designed and constructed cysK-C by substituting serine for the only cysteine residue, C43, found adjacent to the active site K42 (Supplementary Figs S3A and S3B). However, the cysK-C gene was not able to restore the growth of our ΔcysKΔcysM auxotrophic E. coli strain (data not shown) and we subsequently focused on cysE and cysM variants.

### Structural analysis of CysE and CysM proteins

**Figure 3:**
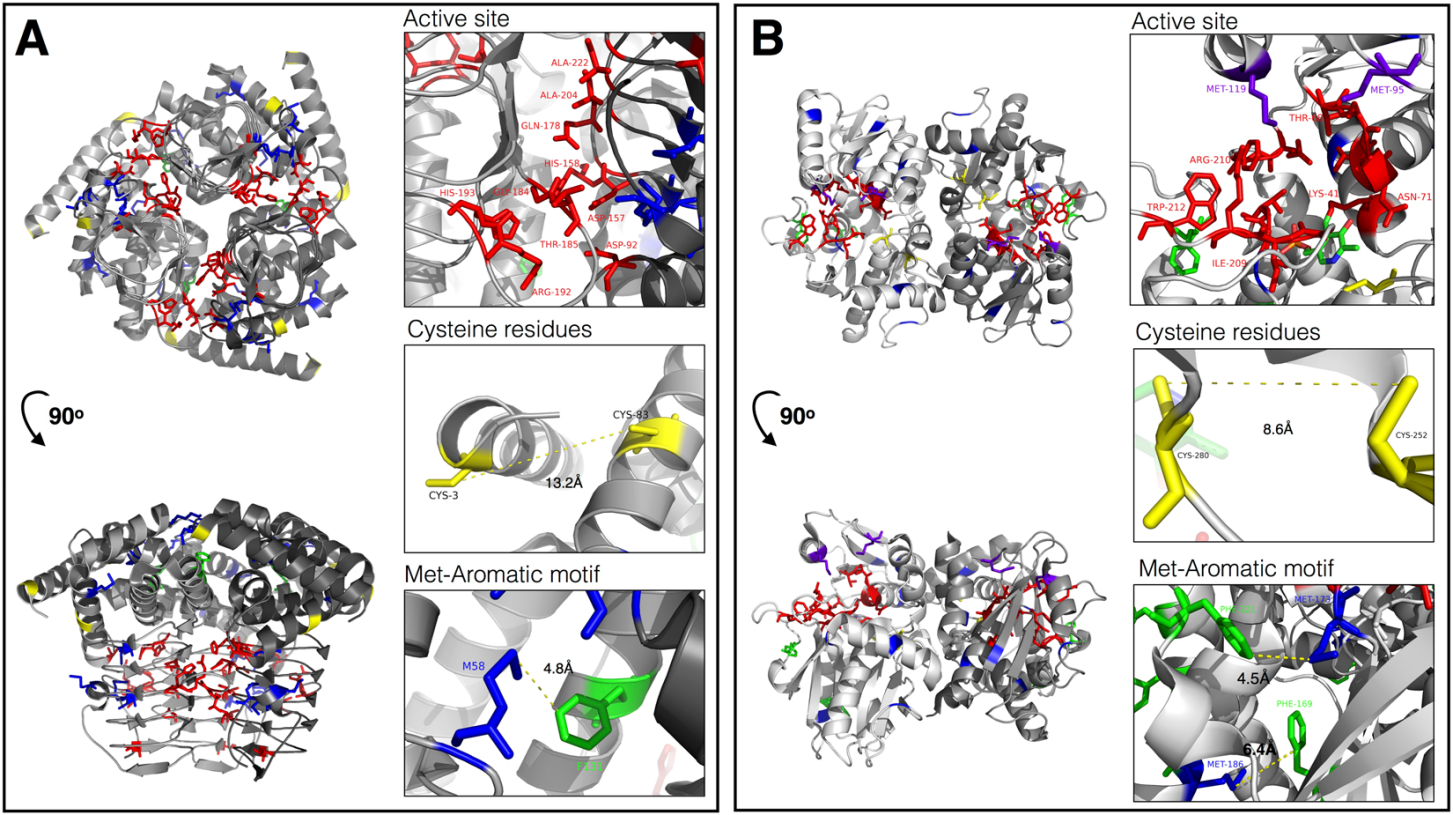
Key residues highlighted on the crystal structure model of CysE and CysM proteins. Cartoon diagram of the trimer (**A**) *E. coli* serine acetyltransferase protein (CysE, PDB ID: 1T3D) and dimer (**B**) *E. coli* O-acetylserine sulfhydrylase (CysM, PDB ID: 2BHS) are shown in gray scheme with each monomer in different exposure. The active site residues (red), substituted cysteine (yellow) and methionine (blue or purple) residues are highlighted in the left diagram. Methionines displayed in purple participate as an active site residue while methionines in blue are not part of the active site. Panels on the right represent the active center (top), the two closest cysteines (middle), and an example of methionine-aromatic motifs (bottom) found within the protein structures of CysE and CysM. Distances between each amino acid residue are denoted in angstroms.

To explore the potential effects of these amino acid substitutions in the context of protein structure, we analyzed the crystal structures of the native CysE and CysM proteins. The quaternary structure of CysE (PDB ID: 1T3D) is a dimer of trimers, with each of the trimer subunits creating three separate active sites (Fig. 3A). No cysteine-cysteine disulfide bridges are expected because the closest cysteine residues, C3 and C83, have a separation of 13.2 Å that is significantly greater than the accepted bridge length of 2.3 Å^18^. In addition, several methionine-aromatic ring motif interactions were observed, with a separation of ~5 Å, that may stabilize protein structure^19^. One such interaction was between the M58 residue and the F131 residue (Fig. 3A).

The quaternary structure of CysM (PDB ID: 2BHS) consists of two monomers that come together to form a dimer with two actives sites (Fig. 3B). Like CysE, CysM does not have any predicted cysteine-cysteine disulfide bridges. The closest two cysteine residues, C252 and C280, have a separation of 8.6 Å, which is greater than the 2.3 Å typical bridge length. CysM also has several methionine-aromatic ring motifs (e.g., M173 and F221) (Fig. 3B).

By performing a search of related sequences and protein structures using HHblits, we obtained multiple sequence alignments (MSAs) for CysE and CysM. For CysE, a MSA with 1,918 sequences was obtained. This MSA had a probability of 100.0, E-value of 3 × 10^−146^, P-value of 3 × 10^−151^, score of 828.2, 0.0 SS, 273 Cols, 1–273 Query HMM, and 42–314 (314) template HMM. For CysE, a MSA with 124 sequences was obtained with a probability of 100.0, 1 × 10^−160^ E-value, 9 × 10^−166^ P-value, 907.8 score, 0.0 SS, 302 Cols, 1–303 Query HMM, and 4–315 (315) template HMM. The consensus amino acid for each residue in CysE and CysM was determined and displayed by HHblits (Supplementary Fig. S2).

### Auxotrophy of cysteine-dependent *E. coli* knockout strains

To determine if the cysteine biosynthesis enzymes lacking cysteine and methionine can enable biosynthesis of cysteine, we obtained the K-12 *E. coli* single knockout strain Δ*cysE* from the Keio collection^20^. We then constructed a double knockout strain, Δ*cysK*Δ*cysM*, by deleting the two homologous O-acetylserine sulfhydrylase genes, which are responsible for converting O-acetylserine to L-cysteine. We tested the auxotrophy of Δ*cysE* and Δ*cysKΔcysM* knockout strains by observing growth on Luria Broth (LB) medium, M9 + glucose medium and M9 + glucose medium supplemented with 0.5 mM L-cysteine. *E. coli* colonies were observed on LB and M9 + glucose medium containing cysteine after 24 and 48 h of incubation respectively, while no growth was observed on M9 + glucose minimal medium after 72 h (and even after seven days), indicating that the knockouts strains are cysteine-dependent auxotrophs (Supplementary Fig. S4).

### Cysteine-free proteins rescue cysteine-dependent knockout strains

**Figure 4:**
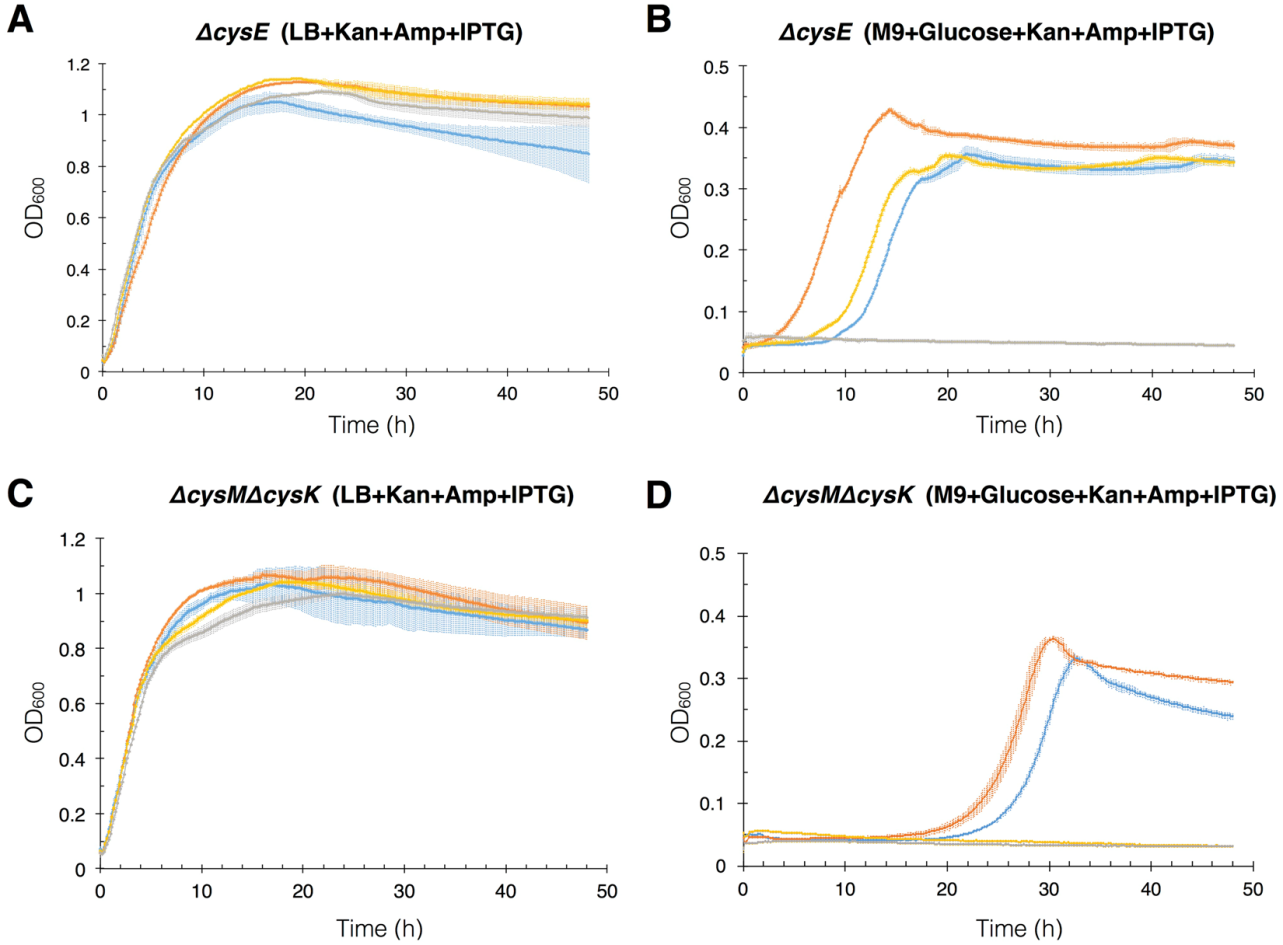
Synthetic c*ysE* and *cysM* gene transformants display recovery of CysE function without cysteine and methionine and CysM function without cysteine. (**A**) *E. coli ΔcysE* competent cells were transformed with positive control *cysE*, two *cysE* variants *cysE-C/cysE-CM* cloned into the multiple cloning site of pUC19 plasmid, and original pUC19 encoding N-terminal fragment of *lacZ*α as a negative control. (**B**) *E. coli ΔcysMΔcysK* competent cells transformed with positive control c*ysM*, two *cysM* variants *cysM-C*/*cysM-CM* in pUC19 plasmid, and original pUC19 encoding N-terminal fragment of *lacZ*α as a negative control. Cells were plated on M9 + glucose medium with 0.4 mM IPTG, 50 μg/ml kanamycin, and 100 μg/ml ampicillin and incubated at 30 °C for 72 h.

Rescue experiments were conducted by transforming three *cysE* variants *(cysE, cysE-C* and *cysE-CM*) into the *ΔcysE* strain and three *cysM* variants (*cysM, cysM-C* and *cysM-CM*) into the Δ*cysK*Δ*cysM* strain. Wild-type *cysE* and codon optimized *cysM* served as positive controls, whereas pUC19 vector with *lacZ* gene was used as a negative control. Transformants were plated on M9 + glucose with ampicillin (Amp) and kanamycin (Kan), and isopropyl-β-D-thiogalactoside (IPTG) supplemented and incubated at 30 °C. Colonies were observed on plates with the native *cysE* and *cysM* transformed cells and the synthetic c*ysE-C, cysE-CM* and *cysM-C* transformed cells. These results suggest the recovery of *cysE* and *cysM* function using cysteine-free enzymes. However, no growth was observed for plates with c*ysM-CM* transformed cells. Negative controls, with *lacZ* transformed cells, also showed no growth (Fig. 4). Initial transformation efficiencies for fully synthesized gene variants were low, possibly due to the errors during gene assembly or PCR amplification. Therefore, to confirm that the cysteine-dependent auxotrophs were rescued by the transformed synthetic genes, we isolated single colonies of c*ysE-C, cysE-CM* and *cysM-C* from M9 + glucose plates and expanded the colonies in liquid LB + Amp culture overnight at 37 °C. The plasmids from these cells were then extracted for sequencing. Sequencing results indicated that all three genes recovered from rescued cells encoded the specific sequences as designed, with no reverse mutations. Likewise, we isolated *cysM-CM* transformed colonies from a LB + Amp plate (since no cells grew on M9 + glucose) and confirmed that the plasmid insert sequence was exactly the *cysM-CM* sequence as designed. The isolated plasmids were further re-transformed into new Δ*cysE* and Δ*cysK*Δ*cysM* knockout cells and plated on M9 + glucose + Amp + Kan + IPTG at 30 °C along with the positive and negative controls. Colonies were again observed after 72 h, indicating that the observed rescue phenotype is specific to the synthetic genes encoded by the plasmids (Fig. 4).

### Growth curve analysis reveals shorter lag phase in cysteine-free gene transformants

**Figure 5:**
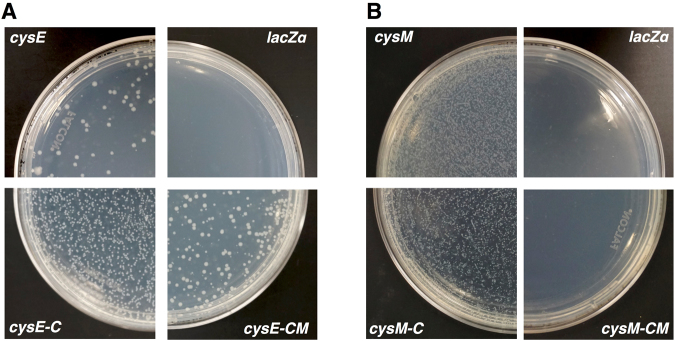
Growth curve of re-transformed auxotrophic *E. coli* strains in LB and M9 + glucose media. Each panel represents growth of cysteine-dependent *E. coli* auxotrophs rescued by wild type enzymes: CysE or CysM (blue), cysteine-free enzymes: CysE-C or CysM-C (orange), cysteine- and methionine-free enzymes: CysE-CM or CysM-CM (yellow), and LacZα protein expressed from original pUC19 plasmid (gray). Growth curve was monitored every 10 minutes at OD 600 nm using 96-well plate reader. Standard deviations of the growth curves are displayed as calculated from triplicates.

To test whether the synthetic genes functioned better, worse, or the same as the modern genes that they had replaced, we performed comparative growth curve assays in liquid LB and M9 + glucose media for *ΔcysE* transformants using c*ysE*, c*ysE-C*, *cysE-CM*, and LacZα (empty pUC19) as rescue genes (Fig. 5A and B), and *ΔcysKΔcysM* transformants using *cysM*, *cysM-C*, *cysM-CM*, and LacZα (empty pUC19) as rescue genes (Fig. 5C and D). Cells were pre-cultured in LB + Amp and washed three times with 0.9% saline solution to remove all nutrients before inoculation to LB or M9 + glucose medium supplemented with ampicillin, kanamycin and IPTG for gene expression. Cell growth was monitored in a 96-well plate for 48 h to observe how each synthetic gene affects distinct phases (lag, exponential, and stationary) during continuous culture. As predicted, in the LB + IPTG condition, overall consistent growth was observed for all transformants without any noticeable change in the lag phase. Whereas, when grown in M9 + glucose medium, auxotrophic strains showed different durations of lag phase. Strains rescued by cysteine-free enzymes CysE-C and CysM-C both experienced relatively shorter lag phase and slightly faster growth rate compared to the strain rescued by wild-type enzymes (Table 1).

**Table 1:** Growth characteristics of rescued auxotrophic *E. coli* strains.

Growth condition	LB + Kan + Amp + IPTG								M9 + Glucose + Kan + Amp + IPTG				
*E. coli* type strain	*ΔcysE*				*ΔcysMΔcysK*				*ΔcysE*			*ΔcysMΔcysK*	
Rescue gene variants	*cysE*	*cysE-C*	*cysE-CM*	*LacZ*α	*cysM*	*cysM-C*	*cysM-CM*	*LacZ*α	*cysE*	*cysE-C*	*cysE-CM*	*cysM*	*cysM-C*
Lag phase (h)	1.16	1.16	1	0.66	0.66	0.83	0.83	1	12	5.5	10.33	27.33	24.83
Log phase (h)	4.33	8	5.33	5	5.33	4.83	4	4.66	4.66	7	4.33	4.66	4.66
Maximum growth rate (h^−1^)	0.29	0.25	0.31	0.29	0.31	0.35	0.32	0.27	0.063	0.068	0.069	0.069	0.076
Maximum generation time (min)	25.5	29.6	23.8	25.5	23.8	21.1	23.1	27.4	117.3	108.7	107.1	107.1	97.2

### Recombinant cysteine-free CysE and CysM variants retain enzymatic activity

To validate the short lag phase observed with cysteine-free protein variants, we conducted cloning, expression and purification of recombinant CysE, CysE-C, CysE-CM, CysM, CysM-C, CysM-CM proteins to confirm their enzymatic activity *in vitro*. In addition, we included a new variant CysM-CM2, a cysteine-free CysM with two methionine substitutions (M95I and M119L) in the active center only, to see if these substitutions alone diminish the function of CysM enzyme. A total of seven FLAG-tagged recombinant proteins were expressed in *E. coli*, and cell lysate fraction was collected to further purify the target protein using Anti-FLAG M2 magnetic beads. As a result, CysE, CysE-C, CysE-CM, CysM, CysM-C, CysM-CM2 proteins were efficiently expressed and purified. However, we were not able to obtain CysM-CM, likely due to instability of the protein (Supplementary Fig. S6B).

**Figure 6:**
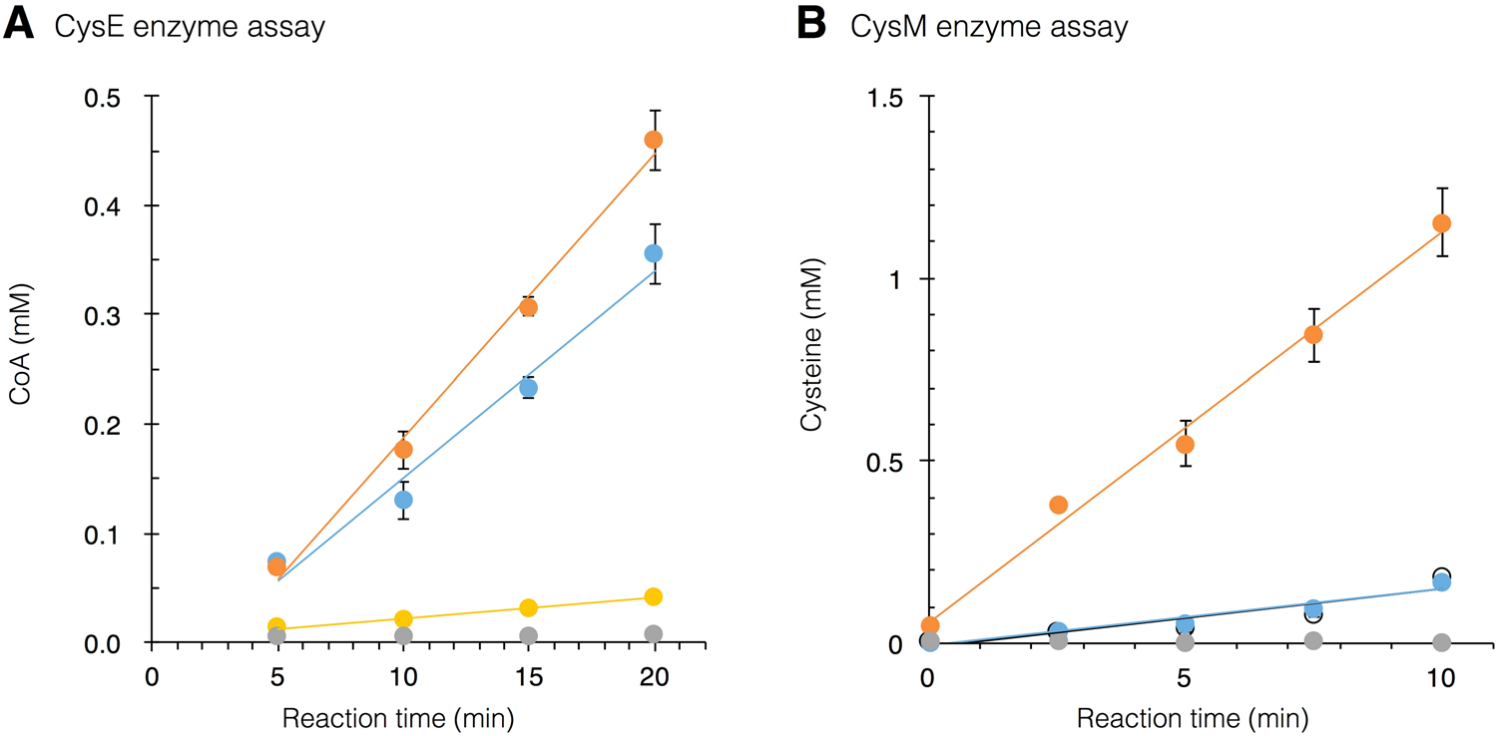
Spectrophotometric assay of recombinant CysE and CysM protein variants. (**A**) Enzymatic activities of CysE recombinant protein variants: CysE (blue), CysE-C (orange), CysE-CM (yellow) are measured as a conversion of L-serine to CoA using Ellman’s reagent reaction at four time points (t = 5, 10, 15, and 20 minutes). (**B**) Enzymatic activities of CysM recombinant protein variants CysM (blue), CysM-C (orange), CysM-CM2 (open circle) are measured as a conversion of O-acetylserine to L-cysteine using Ninhydrin reaction at five time points (t = 0, 2.5, 5, 7.5 and 10 minutes). Protein negative control samples are represented as gray circle. Error bars represent the standard deviation as derived from the triplicates for each sample. Linear regression is presented to calculate the enzymatic activity of each protein variant.

Spectrophotometric assay was conducted to measure the enzymatic activity for recombinant CysE and CysM variants by monitoring the increase in absorbance assayed with Ellman’s Reagent or Ninhydrin, with respect to CoA and L-cysteine production respectively (Fig. 6). Among CysE variants, CysE-C showed significantly higher enzymatic activity (35.1 μmol/min/mg) to the wild-type, whereas CysE-CM only expressed approximately 10% of the activity of the wild-type (Fig. 6 and Table 2). Among CysM variants, CysM-C showed significantly higher activity (21.6 μmol/min/mg) to the wild-type, while CysM-CM2 maintained a comparable level of activity to the wild-type (Fig. 6B and Table 2).

**Table 2:** Enzymatic activity of recombinant CysE and CysM protein variants.

Protein variants	*cysE*	*cysE-C*	*cysE-CM*	*cysM*	*cysM-C*	*cysM-CM2*
Enzymatic activity (μmol/min/mg)	25.5	35.1	2.6	3.16	21.4	3.20

## Discussion

The focus of this study was to address the chicken-and-egg problem of cysteine biosynthesis by introducing synthetic cysteine-free enzymes, and enzymes both cysteine-free and methionine-deficient, to replace their wild-type counterparts in modern *E. coli*. The underlying hypothesis is that primordial polypeptides made from the abiotic subset of modern proteinogenic amino acids can serve as ancestral metabolic catalysts that, over evolutionary time scales, diversify and increase to the repertoire of amino acids found today. The most parsimonious explanation is that cysteine was produced during the evolution of the metabolic network, sustained by early enzymes emerging from common amino acids in prebiotic settings^3^. As such, we took a first step toward recreating these early enzymes through protein engineering, and observed functionality through strain rescue, growth performance, and *in vitro* enzyme activity assay.

We engineered constructs for CysE, CysK, and CysM genes, and proceeded to conduct growth curve and protein activity analysis on CysE and CysM mutants. However, we were unable to rescue *E. coli* expressing reconstructed CysK lacking cysteine (CysK-C). The failure of the CysK-C mutant is likely due to the location of the single cysteine residue existing adjacent to the active site of the enzyme, and closely associated with the binding of pyridoxal 5′-phosphate (PLP). Upon further investigation, we also found that a majority of orthologs outside *E. coli* do not contain cysteine in this location, suggesting that aspartic acid could be an effective substitution for future experimentation (Supplementary Fig. S3C).

Experiments using CysE-C and CysM-C enzymes implied that the acetylation of L-serine and the conversion of ester into cysteine can occur in a cysteine-independent manner. Interestingly, growth curve experiments revealed a shorter lag phase of auxotrophs rescued by cysteine-free enzymes CysE-C and CysM-C compared to their wild-type analogs (Fig. 5). Lag phase is a period in which cells adjust their metabolic activity to the new environment and synthesize enzymes and factors necessary for cell division^21^. Since cysteine is no longer a limiting factor for CysE-C and CysM-C protein synthesis, rescued cells can possibly carry out cysteine production immediately, or at least faster than their wild-type counterpart, even under cysteine-deprived condition. In addition, we found that elimination of cysteine also enhanced the activities of both enzymes CysE-C and CysM-C compared to their wild types (Fig. 6 and Table 2), indicating that cysteine plays no unique role in the function of serine acetyltransferase or O-acetylserine sulfhydrylase. The presence of cysteine residues in these enzymes appears not only unnecessary but also sub-optimal. The bioinformatics analysis showing that cysteine is not evolutionarily conserved among CysE and CysM protein orthologs (Supplementary Fig. S2) further supports this scenario.

We additionally pursued reconstructing the same cysteine biosynthesis pathway using synthetic CysE and CysM enzymes lacking not only cysteine but methionine as well, to extend our hypothesis that cysteine production can be carried out by not only cysteine-free enzymes but also by generally sulfur-deprived enzymes. Therefore, in addition to the previous cysteine substitutions, we replaced all methionine residues with either leucine or isoleucine (Met to Leu/Ile), except the initiator N-formylmethionine (fMet) (Fig. 2). The CysE-CM protein, which consists of only 18 types of amino acids, successfully complemented the loss of CysE function, whereas the CysM-CM protein failed to rescue the loss of O-acetylserine sulfhydrylase activity. Since over-expression and anti-FLAG purification of recombinant CysM-CM failed (Supplementary Fig. S6), we conclude that substitution of both cysteine and methionine residues in CysM likely led to overall improper folding and thus aggregation or degradation. As a possible explanation, S/π interactions between the methionine sulfur atom and aromatic amino acids have recently been reported to increase the stability of a protein structure by 1–1.5 kcal/mol^19^. According to the CysM protein structure in Fig. 3B, two methionine-aromatic motifs were observed, which could contribute to stabilizing the coils (173–176 and 217–222) as well as the structural integrity of the α helix (177–187) and the β sheet (168–172) that are in close proximity to each other.

The ability to function without cysteine and methionine in CysE and without cysteine in CysM raises the question of the importance of these residues for enzymatic function in these enzymes. The consensus sequences of aligned CysE orthologs and CysM orthologs show that no strict conservation of cysteine residues was found (Supplementary Fig. S2). Therefore, we assume that cysteine was likely introduced as part of a neutral mutation during the evolution of these enzymes. However, the long lag phase observed by the wild-type CysE under cysteine depleted conditions (Fig. 5), indicates that expression of these cysteine-coding enzymes will be down-regulated in environments deprived of cysteine or precursors. Unlike the cysteine residues, several methionine residues (such as M256 in CysE, and M95 and M119 in CysM) are well conserved among orthologous proteins. The residues M95 and M119 are part of the active center known to interact with sulfate ions^22^. M119 is specifically involved in the dynamic conformational change that narrows the entry channel after substrate binding to allow only the sulfur-bearing small molecules, such as H_2_S and thiosulfate, to enter^20^. Therefore, additional replacement of the two methionine residues (M95I and M119L) from the CysM-C may have altered the configuration of the entry tunnel after OAS-binding. Based on the *in vitro* assay, CysM-CM2 remained functional but has significantly reduced its activity compared to CysM-C, further supporting the above assumption (Fig. 6B and Table 2).

It is becoming more feasible to create proteins with reduced alphabets^23^, and as such, several attempts have been made to create such proteins while maintaining function. Examples include an orotate phosphoribosyltransferase lacking seven types of amino acids (C, H, I, M, N, Q, and W)^24^ and, in an extreme case, a functional chorismate mutase using only nine amino acids^6^. So far, however, there are no reports of proteins with a limited alphabet being directly involved in the biosynthesis of new amino acids. Thus, we believe this work to be the first example of enzymes that lack the amino acid for which they are directly involved in production. While it is extremely unlikely that primordial cysteine synthesis enzymes were similar to the modern enzymes that we see now, the rational engineering of proteins via synthetic biology approaches has provided an instance for how biological pathways could have arisen from proteins utilizing a reduced set of amino acids in combination with an ancestral sequence reconstruction approach^25^. Similar studies should be explored for other amino acid substitutions and biosynthesis pathways.

## Materials and Methods

### Gene design for cysteine-free *cysE* and *cysM* genes

*E. coli* K-12 substrain MG1655 *cysE* (Genbank ID: 732686788) and *cysM* (Genbank ID: 732683705) genes were selected to create synthetic protein products without any cysteine. Gene sequences were modified using the software Geneious 8.0. Codons corresponding to CysE and CysM cysteine residues were substituted with serine codons in the gene sequence. To create the *cysE-C* gene, a total of three cysteine residues—located at amino acid residues 3, 23, and 83—were thus replaced with serine to create the *cysE-C* gene. Likewise, a total of two cysteine residues—located at amino acid residues 252 and 280— were replaced with serine for *cysM-C*. All designed genes were provided by Integrated DNA Technologies (Coralville, IA USA), and codon-optimized using their online service to achieve efficient expression in *E. coli*.

### Design for cysteine-free and methionine-deficient *cysE* and *cysM* genes

Enzymes lacking cysteine and methionine were created based on the *cysE-C* and *cysM-C* gene sequences. The codons for methionine residues were changed to those for either leucine or isoleucine. The *cysE-CM* gene was created by replacing the methionine codons with the leucine codon in all eight residues (26, 48, 58, 77, 155, 201, 254 and 256) of the *cysE-C* gene construct. Similarly, the *cysM-CM* gene was created by replacing eight methionine codons of the *cysM-C* gene with leucine (residues 19, 48, 78, 103, 119, 173, 186, and 241) and three methionine codons with those for isoleucine (87, 95, and 129). All designed genes were provided by Integrated DNA Technologies (Coralville, IA USA), and codon-optimized using their online service to achieve efficient expression in *E. coli*.

### Visualizing the structures of CysE and CysM proteins

CysE (EC2.3.1.30), a 273 amino acid protein, is arranged as a dimer of loosely stacked trimers to carry out serine acetyltransferase function^26^. CysM (EC2.5.1.47) is a 303 amino acid protein that exists as a dimer and is one of the two isozymes that catalyzes the second step of cysteine synthesis^22^. The crystal structures of CysE (PDB ID: 1T3D) and CysM (PDB ID: 2BHS) were obtained from the RSCB Protein Data Bank. PDB files were imported into PyMOL version 1.6.0.0 (Schrodinger, New York, NY). The residues involved with creating the active center pocket (catalytic core and substrate recognition) were identified. The active center residues are 92, 143, 157–158, 178, 184–185,192, 204, 222, and 235 for CysE^27^ and 41, 68–72, 95, 119, 140, 141, 174, 175, 208–210, and 212 for CysM^22^. In addition to modeling, we performed a homology search by iterative HMM-HMM comparison using HHblits Release-2.18.2 (Tübingen, Germany) on the native *E. coli* CysE and CysM amino acid sequences. The multiple sequence alignment was performed with the following settings: FASTA alignment format, fraction of gaps <50%, uniprot20_Mar12 HMM database, 1 max iteration, global alignment mode, realign with MAC checked, and 0.0 MAC realignment threshold.

### Gene synthesis, cloning, and transformation

Five different gene constructs were synthesized using the gBlocks Gene Fragments technology (Integrated DNA Technologies, Coralville, IA USA). The gene constructs are *cysE-C* (*cysE* with cysteine replaced), *cysE-CM* (*cysE* with cysteine and methionine replaced), *cysM* (codon-optimized *cysM* gene), *cysM-C* (*cysM* with cysteine replaced), and *cysM-CM* (*cysM* with cysteine and methionine replaced). For each gene construct, we added sequences containing HindIII and XhoI restriction sites at the 5′ and 3′ ends. PCR amplification of the gene construct was carried out with Q5 High-Fidelity Master Mix (New England Biolabs Inc., Ipswich, MA, USA) using the forward tag primer (5′-GCTTGCATCGTACGTATCGG-3′), and the reverse tag primer (5′-AGACGTAACGACCAACGCTAG-3′). Amplification was performed in a T100 Thermo Cycler (Bio-Rad Laboratories, Inc., Hercules, CA, USA) using a 30 s initial denaturation at 98 °C, 30 cycles of 10 s denaturation at 98 °C, 15 s annealing at 60 °C, 30 s extension at 72 °C, and a final 2 min extension at 72 °C. PCR products were column purified using the GenCatch PCR Cleanup Kit (Epoch Life Science., Sugar Land, TX, USA) and verified using gel electrophoresis in a 1% (w/v) agarose gel in 1x TAE buffer run at 100 V for 30 min. The resulting gel was stained with GelRed (Thermo Fisher Scientific Inc., Waltham, MA, USA).

For cloning and transformation, both the PCR products and the pUC19 vector (New England Biolabs) were digested using HindIII-HF and XhoI restriction enzymes at 37 °C for 1 h in 1x CutSmart buffer (New England Biolabs). The digested products were then cleansed of extraneous DNA using the MinElute Reaction Cleanup Kit (QIAGEN, Germantown, MD). Fifty nanograms of pUC19 and 50 ng of digested PCR products encoding the gene construct were mixed with ElectroLigase mix (New England Biolabs) in a final volume of 20 μl in 1x T4 DNA Ligase Reaction Buffer for 1 h. Ligated products were transformed into 50 µl of electrocompetent cells using an Electroporator 2510 (Eppendorf, Hauppauge, NY, USA) with an 1800V pulse. Cells were re-suspended in 500 μl of super optimal broth with catabolite repressor (SOC) medium (20 g tryptone, 0.5 g yeast extract, 0.5 g 10 mM NaCl, 2.5 mM KCl, 10 mM MgCl_2_, and 10 mM MgSO_4_ in 1 L of deionized water adjusted to a final pH 7.0) and incubated for 1 h at 37 °C before plating.

### Auxotrophic *E. coli* strains

We used two cysteine-dependent auxotrophic strains in this study. *ΔcysE* K-12 *E. coli*, a knockout strain (JW3582), was provided through the in-house *E. coli* Keio Knockout Collection, which is a compilation of *E. coli* K-12 single-gene knockout mutants strains with a base genotype of BW25113: Δ(araD-araB)567, lacZ4787(del)::rrnB-3, rph-1, Δ(rhaD-rhaB)568, hsdR514, for all nonessential genes^20^. Since two O-acetylserine sulfhydrylases (CysK and CysM) are present in *E. coli* K-12, and based on the description in the previous reports^28,29^ that the single knockout strain for both genes (*cysK* and *cysM*) formed colonies on minimal medium without cysteine, we further constructed a *ΔcysKΔcysM* double knockout strain. First, the kanamycin marker was removed from the *ΔcysM* knockout strain (JW2414), and then the *ΔcysK*::Km allele was introduced to the resulting strain through P1 transduction. Kanamycin resistant colonies were picked and the target strain, lacking both the *cysK* and *cysM* genes, was selected via PCR using primers specifically designed for *cysK* (JW2407_cysK-up; CCAGTATTGCGATTACCCC and JW2407_cysK-down; TCGATTCAGCTTTGGCTTTT) and *cysM* (JW2414_cysM-up; CGAGCGTTTATTCGTTGGTC and JW2414_cysM-down; TTATCCGGCCTACAAAATCG). Cysteine-dependent auxotrophy of the new *ΔcysKΔcysM* double knockout strain was confirmed on M9 + glucose minimal medium with and without cysteine (Fig. 4).

### Preparation of electrocompetent cells

A single colony each of *E. coli ΔcysE* and *ΔcysKΔcysM* mutants were selected from a fresh LB + Kan plate and inoculated in 10 mL of 2xYT medium (16 g casein digest, 10 g yeast extract and 5 g NaCl per 1 L of deionized water adjusted to final pH 7.0) and grown overnight at 37 °C as a starter culture. The overnight culture was inoculated into 1 L of 2xYT medium and incubated at 37 °C, shaking vigorously until it reached an OD_600_ of 0.6. The culture was then put on ice and centrifuged at 4 °C at 2500 × g for 25 min. The cells were washed two times with 800 mL of 4 °C deionized water. The culture was then re-suspended in 80 mL of 10% glycerol at 4 °C, and pelleted at 4000 × g for 10 min at 4 °C. The supernatant was removed, and 2 ml of 10% glycerol at 4 °C was added to the cells. For storage, the cells were dispensed into 100 µl aliquots, snap frozen in liquid N_2_, and stored at −80 °C for future use.

### Functional screening for cysteine biosynthetic enzymatic function

The pUC19 vectors containing *cysE-C* or *cysE-CM* genes were transformed into electrocompetent *ΔcysE* cells, while vectors *with cysM, cysM-C*, and *cysM-CM* genes were transformed into electrocompetent *ΔcysKΔcysM* cells. One microliter of DNA was added to 79 μl of cells thawed on ice, and the mixture was allowed to incubate at 4 °C for 10 min. The cells were then electroporated at 1800V pulse using an Electroporator 2510 (Eppendorf), followed by incubation in 0.5 mL SOC medium at 37 °C for 15 min. After incubation, 0.5 μl of 0.4 M IPTG was added, and the culture was allowed to incubate for an additional 30 min. The cells were then pelleted at 7500 × g for 90 seconds, washed with 500 μl of M9 + glucose minimal medium, pelleted once again, and re-suspended in M9 + glucose with 0.4 mM IPTG. The transformed cells were then plated on M9 + glucose minimal medium supplemented with 0.4 mM IPTG, 50 μg/ml kanamycin, and 100 μg/ml ampicillin (M9 + glucose + Amp + Kan + IPTG).

The plates were to incubate at 30 °C for one week (168 h) or until colonies formed, whichever occurred first. Colonies that formed on minimal medium were isolated. Two control experiments were also conducted: a positive control consisting of electrocompetent *ΔcysE* cells transformed with *cysE* in pUC19 vector and the other with electrocompetent *ΔcysKΔcysM* cells transformed with codon optimized *cysM* in pUC19 vector. The cells were allowed to incubate at 30 °C, overnight. The second positive control group supplemented the lack of cysteine through growing the knockout cells with empty pUC19 vectors on a fully supplemented media, LB + Amp, and were allowed to incubate at 37 °C overnight. Additionally, negative controls consisting of electrocompetent *ΔcysE* and *ΔcysKΔcysM* cells transformed with empty pUC19 expression vectors, were plated on minimal M9 + glucose + Amp + Kan + IPTG media and allowed to incubate at 30 °C for 168 h.

Colonies were picked and grown overnight in liquid LB + Amp + Kan media. Plasmids from rescued colonies were then purified using the QIAGEN QIAprep Spin Miniprep Kit. These plasmids were sequenced by Elim Biopharmaceuticals, Inc. (Hayward, CA US) with the forward primer 5′-CACTCATTAGGCACCCCAGG-3′ and the reverse primer 5′-GAGACGGTCACAGCTTGTCT-3′. To confirm that the plasmids were responsible for rescuing the knock-out strains, the isolated plasmids and were re-transformed into respective new electrocompetent knockout cells. The cells were plated on M9 + glucose + Amp + Kan + IPTG plates and allowed to incubate at 30 °C for 168 h or until colonies formed, whichever occurred first.

### Growth curve analysis

A total of eight transformants were made: three *cysE* variants *(cysE, cysE-C* and *cysE-CM*) and empty pUC19 plasmid were transformed into the *ΔcysE* strain, and three *cysM* variants (*cysM, cysM-C* and *cysM-CM*) and empty pUC19 plasmid were transformed into the *ΔcysKΔcysM* strain. Single colonies were isolated from each plate of transformants and grown to 0.4–0.6 OD_600_ in LB + Amp + Kan at 37 °C. The cells were pelleted at 7500 × g and washed with 0.9% saline solution three times. Half of the cells were resuspended in LB + Amp + Kan and the other half of the cells were resuspended in M9 + glucose + Amp + Kan. Excluding the transformants with empty pUC19 vector (controls), triplicate 200 µl cultures of each transformant in liquid LB + Amp + Kan + IPTG and M9 + glucose + Amp + Kan + IPTG at 0.05 OD_600_ were added into a 96-well plate respectively. Two 200 μl cultures of *ΔcysE* and *ΔcysKΔcysM* with empty pUC19 vectors and blanks of LB + Amp + Kan + IPTG, and two 200 μl blanks of M9 + glucose + Amp + Kan + IPTG were also added to the 96-well plate. To minimize condensation, the lid of the 96-well plate was coated with Triton X-100 by adding 3–4 mL of 0.05% Triton X-100 in 20% ethanol to the lid and ensuring even coverage of the lid^30^. The solution was then poured off after 30 s, and the lid was allowed to air-dry against a vertical surface. The plate was covered and then placed into a SPECTRAmax Plus384 microplate reader (Molecular Devices, Sunnyvale, CA) and the OD_600_ was taken for 48 hours at every 10 minutes. In order to represent the transition from lag phase to log (exponential) phase, specific *E. coli* growth rate μ was calculated based on the difference between five consecutive OD_600_ measurements, where μ = Δln OD_600_/Δt. Log phase was defined by the period of time in which μ is ≥50% of the maximum μ value (μ_max_) observed for that culture (Supplementary Fig. S5).

### Cloning and recombinant protein purification

Seven new gene constructs (*cysE, cysE-C* and *cysE-CM*, *cysM, cysM-C, cysM-CM* and *cysM-CM2*) harboring FLAG-tag at their protein C-terminal region were designed and synthesized through gBlocks Gene Fragments technology. *cysM-CM2* represents CysM-C with two methionine substitutions M95I and M119L. Synthesized gene constructs were first PCR amplified and sub-cloned into pUC19 plasmid for sequence verification. Monoclonal genes were further PCR amplified and cloned into pCR-Blunt II-TOPO vector using the Zero blunt PCR cloning kit (Thermo Fisher Scientific Inc., Waltham, MA, USA), transformed into One Shot BL21 Star (DE3)pLysS competent cells (Thermo Fisher Scientific Inc., Waltham, MA, USA) and plated for colony formation. Five colonies were randomly picked for each transformed gene construct, and used to inoculate 4 ml liquid LB + Kan pre-culture. After overnight shaking incubation at 37 °C each pre-culture was added to 100 mL fresh liquid LB + Kan. Cultures were incubated at 37 °C for 5 hours, followed by addition of IPTG and reduction of incubation temperature to 30 °C. After additional 15 hours of incubation, cell pellets were collected by centrifugation at 8000 rpm and 4 °C for 5 minutes, and frozen at −80 °C for at least 30 minutes. Protein lysate was harvested by cell pellet lysis through repetitive sonication (2 seconds × 200 times), remaining cell fraction was pelleted by centrifugation, and supernatant collected. Recombinant proteins were purified using Anti-FLAG M2 magnetic beads (Sigma-Aldrich Corporation, St. Louis, MO, USA), with elution using 1:1 3x and 1x FLAG peptide (Sigma-Aldrich Corporation, St. Louis, MO, USA). Majority of the FLAG peptide was removed using size selection Amicon Ultra-0.5 mL 30 K Centrifugal Filters (EMD Millipore Corporation, Billercia, MA, USA). All work was conducted with samples on ice, or refrigerated at 4 °C. For verification, purified proteins were incubated with 4X Bolt LDS Sample Buffer for 10 minutes at 70 °C then separated by SDS-PAGE for 40 min at 170 V using Bolt 4–12% Bis-Tris Plus Gel in a 1X Bolt MES SDS Running Buffer. Bands were visualized with SYPRO Orange Protein Gel Stain. All the kits and consumables related to SDS-PAGE analysis were obtained through Thermo Fisher Scientific Inc., Waltham, MA, USA.

### CysE and CysM activity measurement

CysE activity was measured as a function of the conversion of acetyl-CoA to CoA, through spectrophotometric assay using Ellman’s Reagent^31^. Enzymatic reactions were prepared in a 96-well plate to a volume of 50 μl, containing 50 mM Tris-HCl pH 7.5, 5 mM magnesium chloride, 0.4 mM acetyl-CoA, and 2 mM L-Serine. Reactions were initiated by addition of 37 ng purified CysE protein, and incubated at 37 °C for 5, 10, 15, or 20 minutes. Reactions were terminated by the addition of 50 μl of Stop Solution (50 mM Tris-HCl pH 7.5, 6 M guanidine hydrochloride) at appropriate time points. After all reactions were terminated, 50 μL of Ellman’s Reagent was added to each well, and solutions were incubated at room temperature for 10 minutes. Finally, absorbance was measured at 405 nm using SPECTRAmax Plus384 microplate Reader.

CysM activity was measured as a function of conversion of O-acetylserine (OAS) to L-cysteine, through Ninhydrin spectrophotometric assay^32^. First, enzyme stocks were incubated with PLP-hydrate at 4 °C for 4 hours. Meanwhile, fresh Ninhydrin Reagent 2 was prepared. Enzymatic reactions were prepared in a 96-well plate to a volume of 75 μl, containing 50 mM HEPES, 10 mM OAS, and 4 mM sodium sulfide in water. Reaction mixtures were finalized by addition of enzyme to a final concentration of 5 ng/μl, and initiated by incubated at 37 °C for 0, 2.5, 5, 7.5 and 10 minutes. Reactions were terminated by addition of 15 μl 20% (w/v) trichloroacetic acid at each time point. After all reactions were terminated, 10 μl of 100 mM DTT solution with 17 mM NaOH was added to each well and reactions were incubated at room temperature for 30 minutes to reduce any cystine to cysteine. Subsequently, 100 μl of acetic acid and 100 μl of Ninhydrin Reagent 2 were added to each well. The plate was heated at 90 °C for 10 minutes, and cooled rapidly on ice for 2 minutes. From each well, 90 μl of solution was transferred to the corresponding well in a fresh 96-well plate. Finally, 105 µl of 95% ethanol was added to each reaction product in the fresh plate, and absorbance was measured at 560 nm using SPECTRAmax Plus384 microplate Reader. Enzymatic activities (μmol/min/mg) of CysE and CysM were calculated based on the regression line extrapolated from the measured data points (Fig. 6).

### Data availability

The datasets generated during and/or analyzed during the current study are available from the corresponding author on reasonable request.

## Electronic supplementary material

Supplementary Information
