# Author Correction: Reconstruction of cysteine biosynthesis using engineered cysteine-free enzymes

**DOI:** 10.1038/s41598-019-42196-9

**Published:** 2019-04-12

**Authors:** Kosuke Fujishima, Kendrick M. Wang, Jesse A. Palmer, Nozomi Abe, Kenji Nakahigashi, Drew Endy, Lynn J. Rothschild

**Affiliations:** 1 Earth-Life Science Institute, Tokyo Institute of Technology, Tokyo, 1528550 Japan; 2 Universities Space Research Association, NASA Ames Research Center, Moffett Field, California, 94035 USA; 3 Institute for Advanced Biosciences, Keio University, Tsuruoka, 9970035 Japan; 4 Stanford University Department of Bioengineering, Stanford, California, 94305 USA; 5 Spiber Inc. 234-1 Mizukami, Kakuganji, Tsuruoka, 9970052 Japan; 6 NASA Ames Research Center, Moffett Field, California, 94035 USA

Correction to: *Scientific Reports* 10.1038/s41598-018-19920-y, published online 29 January 2018

This Article contains an error in the order of the figure legends. The legends for Figures 4 and 5 were incorrectly given as those for 5 and 4 respectively.

The legend for Figure 4 should read:

“**Growth curve of re-transformed auxotrophic** ***E. coli*** **strains in LB and M9 + glucose media**. Each panel represents growth of cysteine-dependent *E. coli* auxotrophs rescued by wild type enzymes: CysE or CysM (blue), cysteine-free enzymes: CysE-C or CysM-C (orange), cysteine- and methionine-free enzymes: CysE-CM or CysM-CM (yellow), and LacZα protein expressed from original pUC19 plasmid (gray). Growth curve was monitored every 10 minutes at OD 600 nm using 96-well plate reader. Standard deviations of the growth curves are displayed as calculated from triplicates.”

The legend for Figure 5 should read:

“**Synthetic c*ysE*** **and** ***cysM*** **gene transformants display recovery of CysE function without cysteine and methionine and CysM function without cysteine**. (**A**) *E. coli ΔcysE* competent cells were transformed with positive control *cysE*, two *cysE* variants *cysE-C/cysE-CM* cloned into the multiple cloning site of pUC19 plasmid, and original pUC19 encoding N-terminal fragment of *lacZα* as a negative control. (**B**) *E. coli ΔcysMΔcysK* competent cells transformed with positive control c*ysM*, two *cysM* variants *cysM-C*/*cysM-CM* in pUC19 plasmid, and original pUC19 encoding N-terminal fragment of *lacZα* as a negative control. Cells were plated on M9 + glucose medium with 0.4 mM IPTG, 50 μg/ml kanamycin, and 100 μg/ml ampicillin and incubated at 30 °C for 72 h.”

