# Supplementary material for: Reconstruction of cysteine biosynthesis using engineered cysteine-free enzymes
Source: Sci Rep. 2018 Jan 29;8:1776. doi: 10.1038/s41598-018-19920-y (PMC5788988; doi:10.1038/s41598-018-19920-y)
Supplement: Supplementary file 1 — Supplementary Information [file 41598_2018_19920_MOESM1_ESM.docx]

**Supplementary Information**

**Reconstruction of cysteine biosynthesis using engineered cysteine-free enzymes**

Kosuke Fujishima^†^, Kendrick M. Wang^†^, Jesse A. Palmer, Nozomi Abe, Kenji Nakahigashi, Drew Endy, Lynn J. Rothschild^*^

^†^ These authors contributed equally to this work

* Corresponding Author: lynn.j.rothschild@nasa.gov

**Supplementary Figure S1**

**Fig. S1. PCR amplified *cysE*, *cysE-C*, *cysE-CM*, *cysM*, *cysM-C*, and *cysM-CM* genes.** Expected amplified DNA products for total six *cysE* and *cysM* gene variants. *cysE-WT*: DNA PCR product from NEB 5-alpha competent *E. coli* (High Efficiency) cells; *cysE-C*: DNA PCR product from IDT gBlock; *cysE-CM*: DNA PCR product from IDT gBlock; *cysM-WT*: DNA PCR product from IDT gBlock; *cysM-C*: DNA PCR product from IDT gBlock; *cysM-CM*: DNA PCR product from IDT gBlock. The gel electrophoresis was performed on a 1% (w/v) agarose-TAE gel for 40 min at 100V in 1x TAE solution.

**Supplementary Figure S2**

**Fig. S2. HHblits consensus sequences of multiple sequence alignments for *cysE* and *cysM* display low conservation of cysteine and methionine residues and high conservation of active center residues.** Each alignment is composed of the most commonly substituted amino acid within orthologs (bottom) for each original CysE and CysM protein residue (top). The relative height of each bar represents the prominence of the corresponding amino acid. Between these aligned residues are “|”, “.”, or “+” marks meaning no to little substitution, dissimilar substitution, or similar substitution, respectively. Additionally, active center residues are marked with red diamonds, and residues which were substituted to create *cysE-C*, *cysE-CM*, *cysM-C*, or *cysM-CM* are indicated by black arrows.

**Supplementary Figure S3**

**Fig. S3.** **Key residues in the crystal structure and sequence of CysK protein.** (A) Cartoon diagram of the dimer *E. coli* cysteine synthase A protein (CysK, PDB ID: 5J43) shown in gray scheme with each monomer a different shade. The active site residues (red), cysteine residues (yellow), methionine (blue) and aromatic amino acids (green) are highlighted. Panels on the right represent the active center (top), the two closest cysteines (middle), and an example of methionine-aromatic motifs (bottom) found within the protein structure of CysK. Distances between each amino acid residue are denoted in angstroms. (B) Cysteine residue substitution site for *cysK* gene. The single cysteine residue C43 was replaced with serine. Notably, this site is adjacent to an active site residue S42. (C) HHblits consensus sequence of multiple sequence alignment for CysK protein near the N-terminal active site was visualized using WebLogo3 ^1^. The cysteine residue C43 is highlighted in orange, and active sites in red.

**Supplementary Figure S4**

**
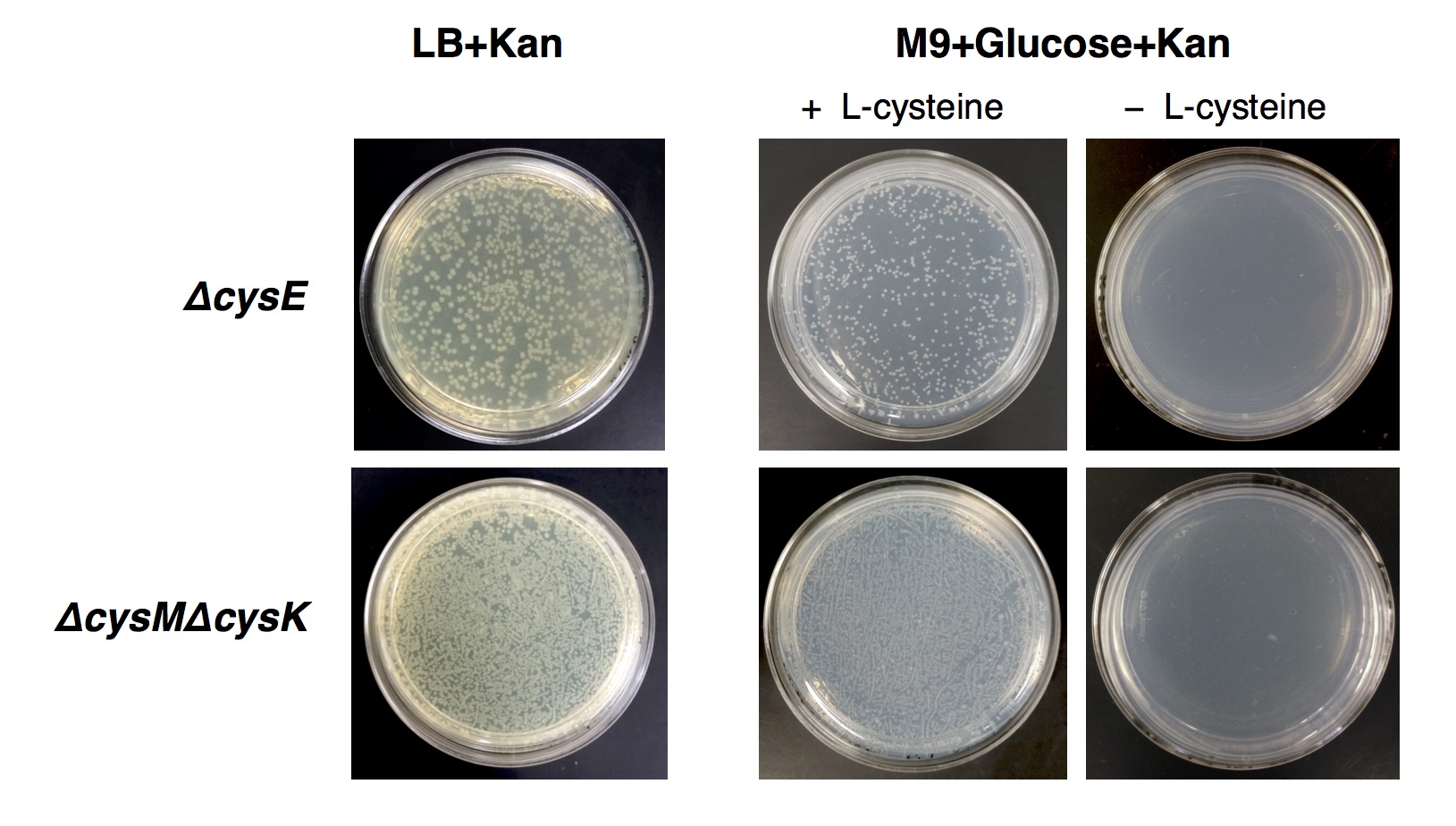
**

**Fig. S4. Cysteine-dependent growth of *ΔcysE* and *ΔcysMΔcysK* *E. coli* knockout strains.** Kanamycin resistant K-12 *E. coli* knockout strains *ΔcysE* and *ΔcysMΔcysK* were plated on LB medium supplemented with 50 µg/ml kanamycin, M9+glucose medium supplemented with 0.5 mM L-cysteine and 50 µg/ml kanamycin, and M9+glucose medium with 50 µg/ml kanamycin. For both strains, colonies were observed after incubation at 37 ℃ for 24 h on LB+Kan, as well as after incubation at 30 ℃ for 72 h on M9+glucose medium supplemented with L-cysteine and kanamycin. In contrast, no growth was observed on M9+glucose medium supplemented only with kanamycin.

**Supplementary Figure S5**

**Fig. S5. Growth curve analysis of *E. coli* knockout strains during the rescue experiment.** The average growth rate μ for each time point is calculated as a difference between five consecutive OD_600_ measurements (Δln OD_600_/ Δt). Growth rate plots were presented with growth curve data obtained from the auxotrophic *E. coli* rescue experiments shown in Figure 5. The start and end points of log phase (dotted line) were defined by the period of time in which growth rate exceeds the 0.5 µ_max_ (red line) observed for that culture.

**Supplementary Figure S6**

**Fig. S6. PCR amplification, transformation and SDS-PAGE of purified FLAG-tagged recombinant CysE and CysM proteins.** A) PCR amplification of seven synthesized *cysE* and *cysM* gene constructs (*cysE, cysE-C,* *cysE-CM, cysM, cysM-C*, *cysM-CM2* and *cysM-CM*) for cloning and protein expression in *E. coli*. B) CysE and CysM protein variants were purified using anti-FLAG M2 magnetic beads then size selected using Amicon Ultra-0.5 mL 30K Centrifugal Filters (see Materials and Methods section). Purified CysE and CysM protein variants (CysE, CysE-C and cysE-CM, CysM, CysM-C, CysM-CM2, CysM-CM) were separated by SDS-PAGE on Bolt 4-12% Bis-Tris Plus Gel along with Novex Sharp Unstained Protein Standard (Thermo Fisher Scientific Inc., Waltham, MA, USA).

**References**

1 Crooks, G. E., Hon, G., Chandonia, J. M. & Brenner, S. E. WebLogo: a sequence logo generator. *Genome Res* **14**, 1188-1190, doi:10.1101/gr.849004 (2004).
